# Effective usage of cationic derivatives of polyprenols as carriers of DNA vaccines against influenza virus

**DOI:** 10.1186/s12985-017-0838-x

**Published:** 2017-09-02

**Authors:** Anna Stachyra, Monika Rak, Patrycja Redkiewicz, Zbigniew Madeja, Katarzyna Gawarecka, Tadeusz Chojnacki, Ewa Świeżewska, Marek Masnyk, Marek Chmielewski, Agnieszka Sirko, Anna Góra-Sochacka

**Affiliations:** 1 Institute of Biochemistry and Biophysics Polish Academy of Sciences, Pawińskiego 5A, 02-106 Warsaw, Poland; 2 Department of Cell Biology, Jagiellonian University, Faculty of Biochemistry, Biophysics and Biotechnology, Gronostajowa 7, 30-387 Kraków, Poland; 3 Institute of Organic Chemistry Polish Academy of Sciences, Kasprzaka 44/52, 01-224 Warsaw, Poland

**Keywords:** Adjuvant, Vaccine delivery, Lipofection, Immunization, Humoral response

## Abstract

**Background:**

Cationic derivatives of polyprenols (trimethylpolyprenylammonium iodides – PTAI) with variable chain length between 6 and 15 isoprene units prepared from naturally occurring poly-*cis*-prenols were tested as DNA vaccine carriers in chickens and mice. This study aimed to investigate if PTAI could be used as an efficient carrier of a DNA vaccine.

**Methods:**

Several vaccine mixtures were prepared by combining different proportions of the vaccine plasmid (carrying cDNA encoding a vaccine antigen, hemagglutinin from H5N1 influenza virus) and various compositions of PTAI. The vaccines were delivered by intramuscular injection to either chickens or mice. The presence of specific antibodies in sera collected from the immunized animals was analyzed by enzyme-linked immunosorbent assay (ELISA) and hemagglutination inhibition (HI) test.

**Results:**

The mixtures of PTAI with helper lipids, such as DOPE (1,2-dioleoyl-sn-glycero-3-phosphatidylethanolamine), DC-cholesterol [{3ß-[N-(N′,N′-dimethylaminoethane)-carbamoyl] cholesterol} hydrochloride] or DOPC (1,2-dioleoyl-sn-glycero-3-phosphatidylcholine) induced strong humoral response to the antigen encoded by the DNA vaccine plasmid.

**Conclusion:**

The animal immunization results confirmed that PTAI compositions, especially mixtures of PTAI with DOPE and DC-cholesterol, do work as effective carriers of DNA vaccines, comparable to the commercially available lipid transfection reagent.

## Background

In the past two decades considerable development of DNA vaccine technology has been observed. This technology has emerged as a promising alternative to traditional vaccines and can be applied for therapy of human and animal infections, cancers, allergies or autoimmune disorders. The main advantages of DNA vaccines are: (i) rapid and relatively inexpensive mass production and (ii) simplicity of development, modification, formulation and preparation. DNA vaccines can induce both humoral and cellular responses highly specific to the chosen antigen, of which the structure and posttranslational modification are like those in natural infection. The mechanism by which a DNA vaccine works has been presented in several recently published reviews, for example [1–3]. Briefly, a DNA vaccine which consists of an animal expression vector carrying a sequence encoding a selected antigen and DNA carrier is injected into the tissue, resulting in somatic and/or antigen-presenting cell (APC) transfection. Then, vaccine DNA translocates to the cell nucleus where the transgene is transcribed by host enzymes followed by translation in the cytoplasm. After processing, the antigenic peptides bind to MHC class I and class II and are presented by APC to activate naïve T cells. Alternatively, secreted antigenic proteins can be processed to activate B cells for antibody production. In comparison to live attenuated or inactivated vaccines the immunogenicity of DNA vaccines, especially in humans and large animals, is usually lower. Improvement of both the expression level of an antigen and the efficiency of transfection are essential for elevation of DNA immunization efficacy. Thus various modifications of expression cassettes (e.g. codon optimization, introducing of various regulatory elements, other changes in the antigen coding sequence), biological and biochemical adjuvants, different carriers and vaccination routes and other strategies were widely investigated [4].

One of the commonly used methods of delivery of DNA vaccines is the application of lipofectants, i.e. cationic lipids as DNA vehicles, which are able to spontaneously interact with negatively charged nucleic acids forming lipoplexes (lipid–nucleic acid complexes). Non-specific interactions with the surface of cellular membranes stimulate the uptake of lipoplexes into the cell via endocytosis, while the presence of helper lipids (e.g. neutral lipids, such as DOPE [1,2-dioleoyl-sn-glycero-3-phosphatidylethanolamine], DC [{3ß-[N-(N′,N′-dimethylaminoethane)-carbamoyl] cholesterol} hydrochloride] or DOPC [1,2-dioleoyl-sn-glycero-3-phosphatidylcholine]) modifies the properties of lipoplexes and facilitates DNA release from endosomes.

A broad prospect for applications of cationic lipids as DNA vaccine carriers and adjuvants is the basis for extensive studies aimed at elaboration of effective and safe lipid vehicles [5, 6]. Numerous studies have confirmed the positive effect of lipid adjuvants [7, 8]; however, so far the ultimate lipid component of vaccines remains to be discovered. This might be the result of a broad diversity of vaccines and their administration protocols, divergent diseases and responsive mechanisms of the organisms and, finally, numerous side effects caused by vaccination. An additional level of complexity in the search for optimal vaccine carriers and adjuvants is added by the diversity of immunization mechanisms. All these issues result in a complex relationship between the components of the vaccine and their safety and efficacy. Cationic derivatives of polyprenols, named either PTAI (trimethylpolyprenylammonium iodides) or AP (amino-prenols), have been already recognized as lipofectants and their usage in in vitro cell transfection described [9–11].

In this work we investigated if PTAI with diverse length polyprenyl chains (6–15 units) can be used as effective carriers of DNA vaccines. In immunization experiments we used two animal models, chickens and mice. Animals were immunized with the DNA vaccine encoding hemagglutinin (HA) from influenza virus H5N1 which was developed and successfully used by us previously [12–14]. The immunogenicity of the DNA vaccine was evaluated by enzyme-linked immunosorbent assay (ELISA) and hemagglutination inhibition (HI) test. Comparison of the results obtained with vaccine mixtures containing several compositions of PTAI is presented and discussed.

## Methods

### Vaccine plasmid

The K3 plasmid is a recombinant expression vector pCI (Promega) carrying under control of the cytomegalovirus promoter the cDNA encoding the full length hemagglutinin (without cleavage site between subunits HA1 and HA2) from the highly pathogenic H5N1 influenza virus strain A/swan/Poland/305-135 V08/2006 (clade 2.2.) [12]. After propagation in *Escherichia coli* (DH5α strain), plasmid DNA was isolated using the NucleoBond® PC 10000 EF endotoxin-free giga purification kit (Macherey-Nagel) which ensures endotoxins level < 0.1EU/μg DNA. Purified DNA was dissolved in PBS buffer, pH 7.4.

### Preparation of PTAI compositions

**Fig. 1:**
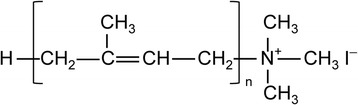
Structure of trimethylpolyprenylammonium iodides (PTAI). PTAI with diverse length of polyprenoid chains were investigated, respectively *n* = 6-8 (mixture), *n* = 11, *n* = 15 and *n* = 10-14 (mixture)

Four types of PTAI preparation with different length polyprenoid chains were used in this work: (i) PTAI-11 containing 11 isoprenoid subunits, (ii) PTAI-15 containing 15 isoprenoid subunits, (iii) PTAI-6-8 and (iv) PTAI-10-14, containing a mixture of polyprenols with chain lengths from 6 to 8 subunits and from 10 to 14 subunits, respectively (Fig. 1). The PTAI were obtained as previously described [10, 11], (patent PL 2012, B1 211824, Patent Office of the Republic of Poland). PTAI and helper lipids (DOPE, DC-cholesterol and DOPC were dissolved in 99.8% ethanol). They were mixed in appropriate ratios in order to achieve the following molar ratios in the mixture: The following five compositions containing PTAI and helper lipids in the indicated molar ratios were prepared: PTAI-6,7,8 + DOPE – 1,5:1; PTAI-11 + DOPE + DC-cholesterol – 1:1:1; PTAI-11/DOPE/DC-cholesterol/DOPC - 1 : 1 : 1 : 1, PTAI-15/DOPE/DC-cholesterol/DOPC - 1 : 1 : 1 : 1 and PTAI-10-14/DOPE/DC – 1:1:1. Then the obtained mixtures were mixed with endotoxin tested DMEM F-12 HAM cell culture medium without additions of serum and antibiotics and intensely vortexed for 3 min. Ethanol content in the mixtures was: PTAI-11/DOPE/DC cholesterol/DOPC – 21,3%, PTAI-15/DOPE/DC cholesterol/DOPC – 15,6%, PTAI-6-8/DOPE – 11,4%, PTAI-11/DOPE/DC-cholesterol – 20,7%, PTAI-10-14/DOPE/DC-cholesterol – 20,7%. The compositions were stored at 4 °C up to 5 days.

### Preparation of vaccine mixtures and animal vaccination

The vaccine mixtures were prepared by combination of the K3 plasmid with the prepared PTAI compositions in different PTAI : DNA ratios (w/w), depending on the DNA dose used in the experiment which is indicated separately. Before injections PTAI compositions were mixed with plasmid DNA and incubated for 30 min at room temperature. The mixtures of K3 plasmid DNA (amounts corresponding to the K3 amounts used with PTAI) and Lipofectin® Transfection Reagent (Invitrogen™) served as pertinent controls. These mixtures were prepared by mixing DNA with this carrier at a ratio (w:v) of 6 : 1, as described previously [12]. Briefly, Lipofectin was initially diluted in PBS, mixed with DNA and incubated for at least 10 min at room temperature, according to manufacturer protocol.

One vaccine dose for chickens contained either 125 μg or 62.5 μg of plasmid DNA in a final volume of 160 μl or 100 μl, respectively, while one vaccine dose for mice contained 10 μg of plasmid DNA in a final volume of 50 μl.

Two types of chicken: broilers (Ross 308) and laying hens (Rosa 1) were immunized intramuscularly in the breast muscle twice on the 7th and the 21st day of life. Blood samples were taken from the wing vein 14, 21 and 28 days after initial immunization. Mice (BALB/c) were immunized intramuscularly twice on the 35th and 49th day of life. Blood was collected at days 14, 21 and 28 days after initial immunization. The group sizes (*n* = 5-10 for the tested groups and *n* = 2-5 for the control groups) are indicated in the respective figures.

Details of the PTAI compositions and vaccine doses used in the three immunization experiments are indicated in Table 1.

**Table 1:** Details of the tested vaccine mixtures

Animal model	Group	PTAI compositions or Lipofectin	Molar ratio PTAI: helper lipids	DNA dose	Ratio^a^
Chicken-broilers	1	PTAI-6-8 with DOPE	1.5:1	125 μg	0.9:1
	2	PTAI-11 with DOPE/DC	1:1:1	125 μg	0.8:1
	3	PTAI-11 with DOPE/DC/DOPC	1:1:1:1	125 μg	1.3:1
	4	PTAI-15 with DOPE/DC/DOPC	1:1:1:1	125 μg	1.6:1
	5	Lipofectin	n.a	125 μg	1:6
	6	PTAI-6-8 with DOPE or Lipofectin^b^	1.5:1	125 μg	0.9:1 or 1:6
Chicken-layers	1	PTAI-11 with DOPE/DC	1:1:1	125 μg	0.8:1
	2	PTAI-11 with DOPE/DC	1:1:1	62.5 μg	1.6:1
	3	PTAI-11 with DOPE/DC	1:1:1	62.5 μg	0.8:1
	4	Lipofectin	n.a	125 μg	1:6
	5	Lipofectin	n.a	62.5 μg	1:6
	6	None	n.a	125 μg	n.a
	7	None	n.a	62.5 μg	n.a
	8	Lipofectin	n.a	125 μg	1:6
Mice	1	Lipofectin	n.a	10 μg	1:6
	2	PTAI-10-14 with DOPE/DC	1:1:1	10 μg	0.8:1
	3	PTAI-10-14 with DOPE/DC	1:1:1	10 μg	0.8:1

Vaccine mixtures were prepared by mixing the K3 plasmid with PTAI compositions or Lipofectin. Groups 6 and 7 (chicken-layers) were immunized with vaccine containing the K3 plasmid in PBS, pH 7.4 without any carriers. ^a^ratio for PTAI:DNA (w:w) or for Lipofectin:DNA (v:w) in vaccine doses is indicated; ^b^in control group empty pCI vector was used with PTAI-6-8 (*n* = 3) or Lipofectin (*n* = 2); n.a not applicable

### ELISA assay and HI test

The ELISA assay detecting anti-H5 IgY in chicken sera and anti-H5 IgG in mouse sera as well as HI test were performed as described previously [12, 13]. Purified recombinant H5 HA (A/swan/Poland/305-135 V08/2006) derived from a baculovirus system (Oxford Expression Technologies, UK) was used for plates coating in ELISA assay. Anti-H5 IgY were detected with goat anti-chicken IgY (Fc-specific)-HRP (Thermo Scientific) while anti-H5 IgG were detected with goat anti-mouse IgG-AP (Sigma-Aldrich). For one dilution ELISA sera were diluted 1:200 (chickens) or 1:100 (mice). For end-point ELISA sera were serially diluted and the end-point titer of the anti-H5 antibodies was determined as the inverse value of the highest serum dilution at which a result higher than the estimated value: background + 3SD (three times the value of the standard deviation) was obtained. HI test were performed according to the OIE standard procedure using the commercially available antigen from low pathogenic H5N2 strain, A/chicken/Belgium/150/1999 (DG Deventer, Netherlands) which share 96% protein sequence similarity with vaccine antigen. Serially diluted sera were incubated with antigen for 25 min, and then incubated with chicken erythrocytes for 30 min. HI titer was defined as the reciprocal of the highest dilution of sera that completely inhibited hemagglutination.

A non-parametric Mann–Whitney U test, which is a component of Statistica 12 software (StatSoft, Poland), was used to evaluate the statistical differences between groups. A value of *p* < 0.05 was considered significant.

## Results

### Chicken immunization

**Fig. 2:**
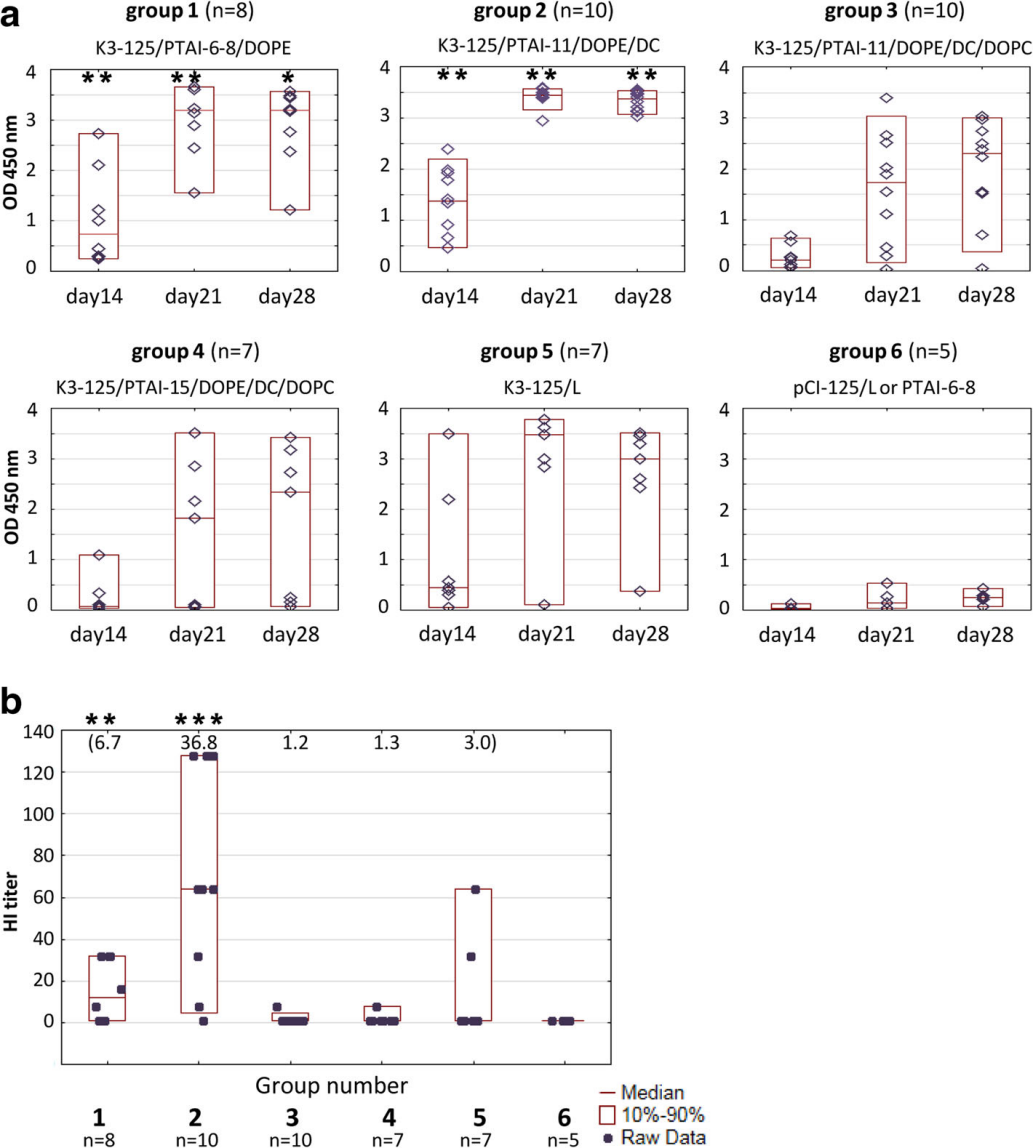
Humoral response of broilers to vaccination. Birds were immunized with a mixture of K3 DNA (125 μg) with the PTAI composition as indicated (PTAI-6-8/DOPE, PTAI-11/DOPE/DC, PTAI-11/DOPE/DC/DOPC, PTAI-15/DOPE/DC/DOPC) or with Lipofectin (L); pCI – group vaccinated with empty pCI vector with PTAI or Lipofectin. **a** ELISA results for individual chickens; raw data (◊), median (—) and the 10th and 90th percentiles (□) are shown for each group; blood collected at indicated day post initial immunization, sera diluted 200-fold. (*) group showed significantly higher ELISA values than K3-PTAI-11/DOPE/DC/DOPC. (**) group showed significantly higher ELISA values than K3-PTAI-11/DOPE/DC/DOPC and K3-PTAI-15/DOPE/DC/DOPC (*p* < 0.05). **b** HI titers in sera of individual chickens on day 28 post immunization. Geometric means for each group are indicated at the top of the graph. (***) The K3-PTAI-11/DOPE/DC group showed a significantly higher HI titer than all other groups (*p* < 0.05)

The groups of chickens, broilers in the first experiment and laying hens in the second, were immunized with two doses of the DNA vaccines containing the K3 plasmid and different PTAI compositions (Table 1). In the first experiment four different PTAI compositions: (i) PTAI-6-8/DOPE, (ii) PTAI-11/DOPE/DC, (iii) PTAI-11/DOPE/DC/DOPC and (iv) PTAI-15/DOPE/DC/DOPC mixed with 125 μg of K3 were tested along with the positive (K3 mixed with Lipofectin® Transfection Reagent) and negative (pCI mixed with one of the carriers) controls. The PTAI : DNA ratios (*w*/w) in vaccine compositions K3-PTAI 6-8/DOPE, K3-PTAI-11/DOPE/DC, K3-PTAI-11/DOPE/DC/DOPC and K3-PTAI-15/DOPE/DC/DOPC were 0.9 : 1, 0.8 : 1, 1.3 : 1 and 1.6 : 1, respectively, while calculated charge ratios PTAI^+^:DNA^−^ or (PTAI + DC)^+^:DNA^−^ were 1:2.3, 1:1.7, 1:1.1 and 1:1.1, respectively. Efficacy of the applied vaccines was assayed by ELISA and HI tests which allowed estimation of the level of induced specific anti-HA antibodies in the collected sera. Independently of the carrier, most chickens immunized with K3 developed a specific antibody against HA, while those vaccinated with the empty vector (pCI, group 6) clearly showed no anti-H5 response (Fig. 2a). All groups immunized with K3 showed an evident increase of humoral response after booster immunization. However, only in the groups vaccinated with K3-PTAI-6-8/DOPE (group 1) and K3-PTAI-11/DOPE/DC (group 2) did 100% of chickens produce specific anti-H5 antibodies. In the K3-PTAI-11/DOPE/DC/DOPC (group 3) and K3-PTAI-15/DOPE/DC/DOPC (group 4) one out of ten and three out of seven immunized chickens, respectively, gave negative results. The strongest responses to vaccination were observed in the K3-PTAI-11/DOPE/DC group. This group had not only the best individual chicken responses in ELISA (Fig. 2a) but also was significantly different from all other groups in the HI test, had the highest scores for individual chickens, and the highest median and geometric mean of HI in comparison to the other groups (Fig. 2b).

**Fig. 3:**
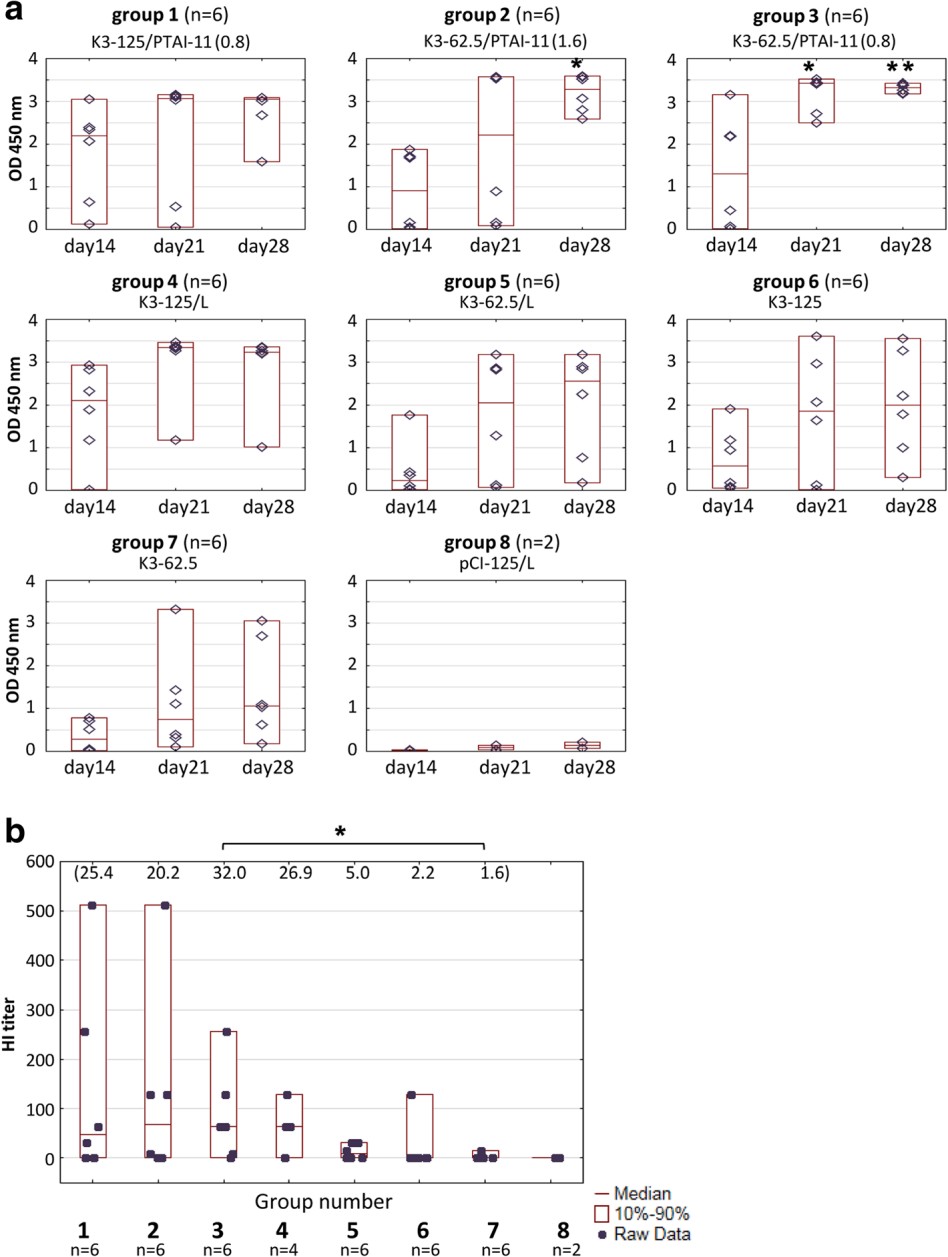
Comparison of humoral response of layers after immunization with different K3 vaccine mixtures. Vaccine doses composed of K3 plasmid alone (125 μg or 62.5 μg) or K3 plasmid with carrier PTAI-11/DOPE/DC (denoted as PTAI) or Lipofectin (L) in different *w*/w ratios indicated in brackets; pCI-125 – group vaccinated with empty pCI vector with Lipofectin. **a** ELISA results for individual chickens; raw data (◊), median (—) and the 10th and 90th percentiles (□) are shown for each group; blood collected at indicated day post initial immunization, sera diluted 200-fold. (*) group showed significantly higher ELISA values than K3-62.5. (**) group showed significantly higher ELISA values than K3-62.5 and K3-62.5/L (*p* < 0.05). **b** HI titers in sera of individual chickens on day 28 post immunization. Geometric means for each group are indicated at the top of the graph. (*) The K3-62.5/PTAI (0.8) group showed a significantly higher HI titer than K3-62.5 (*p* < 0.05)

**Fig. 4:**
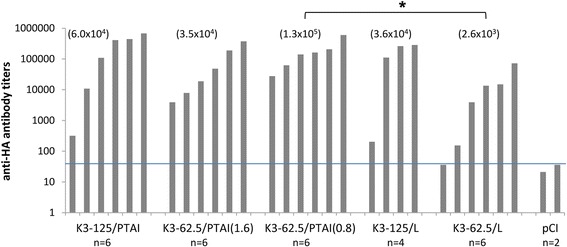
End-point titers of anti-H5 HA antibody in sera collected on the 28th day post immunization. The titer of anti-H5 antibodies was determined as the inverse value of the highest serum dilution at which a result higher than the estimated value: background + 3SD (three times the value of the standard deviation) was obtained. If there was no need for dilutions higher than the standard 200-fold dilution (readout < 1 for the 200-fold dilution), the levels were determined by multiplying the OD value read out in the ELISA test by 200, namely by the inverse value of the serum dilution used in the ELISA test. Geometric means for each group are indicated at the top of the graph. (*) The K3-62.5/PTAI (0.8) group showed a significantly higher anti-HA titer than K3-62.5/L (*p* < 0.05)

Based on one-dilution ELISA and HI tests the PTAI-11/DOPE/DC composition was selected for further trials in the second experiment. Vaccine doses were prepared by mixing PTAI-11/DOPE/DC with 125 μg or 62.5 μg of K3 plasmid DNA (Table 1). Three vaccine compositions were used: (i) K3-125/PTAI (0.8), consisting of 125 μg of DNA per vaccine dose and a PTAI-11 : DNA (*w*/w) ratio of 0.8 : 1, (ii) K3-62.5/PTAI (1.6), consisting of 62.5 μg of DNA and a PTAI : DNA (*w*/w) ratio of 1.6 : 1 and (iii) K3-62.5/PTAI (0.8), consisting of 62.5 μg of DNA and a PTAI-11 : DNA (*w*/w) ratio of 0.8 : 1. Charge ratios calculated as (PTAI + DC)^+^:DNA^−^ for these three vaccine compositions were 1:1.7, 1:0.8, and 1:1.7, respectively. Simultaneously, vaccine compositions containing Lipofectin® instead of PTAI-11/DOPE/DC (K3-125/L and K3-62.5/L, containing 125 μg and 62.5 μg DNA, respectively) and vaccine samples without any carrier (K3-125 and K3-62.5) were prepared. As expected, the anti-H5 levels in the groups immunized without any carrier were lower and much more variable than in the groups immunized with the carriers. Therefore no end-point titer ELISA was performed in the groups immunized without carrier. Interestingly, the PTAI compositions containing the lower dose of plasmid DNA (62.5 μg) seemed to be slightly superior to the vaccine containing the mixture of the same DNA dose with Lipofectin®, the reference carrier (Fig. 3a). This observation is partially supported by statistical analysis, the results of the HI test (Fig. 3b) and the results of endpoint titer evaluation in individual sera collected on day 28 post immunization (Fig. 4), where the highest titers were observed in the K3-62.5/PTAI(0.8) group (geometric mean 1.3 × 10^5^). Moreover, the K3-62.5/PTAI groups, regardless of the PTAI : DNA ratio, had no low responders (chickens with low endpoint titer of anti-HA antibodies in the serum), in contrast to the K3-62.5/L group immunized with the reference carrier.

### Mouse immunization

**Fig. 5:**
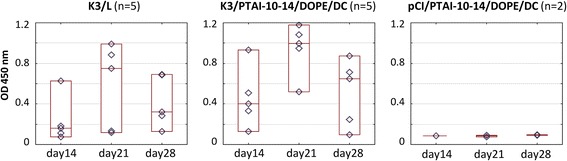
Humoral response of mice after immunization. Vaccine was composed of K3 and one of carrier PTAI-10-14-DOPE/DC-cholesterol or Lipofectin. Raw data (◊), median (—) and the 10th and 90th percentiles (□) are shown for each group; Blood was collected at the indicated day post initial immunization, sera diluted 100-fold; pCI – group vaccinated with empty pCI vector with PTAI

The efficacy of PTAI as a DNA vaccine carrier was also tested in mice (Table 1). Animals were intramuscularly vaccinated with two doses of DNA vaccine. One group had a vaccine consisting of a mixture of K3 (10 μg) and PTAI composition (PTAI-10-14/DOPE/DC) with a PTAI : DNA ratio (w/w) of 0.8 : 1 and calculated charge ratio [(PTAI + DC)^+^:DNA^−^] 1:1.7, while the second group had a vaccine consisting of a mixture of K3 (10 μg) and Lipofectin®. In the negative control K3 was replaced with empty pCI vector. The ELISA results using collected sera demonstrated that the tested PTAI composition is an effective DNA carrier (Fig. 5). However, in this experiment no significant differences between the tested PTAI composition and the control lipid carrier were observed. For example, in both groups the highest levels of anti-HA specific antibodies were observed 1 week after the booster and in both groups low responders were present.

## Discussion

The promising potential of DNA vaccines stimulates the search for efficient, safe and cost-effective carriers of nucleic acids suitable for in vivo application. Such carriers should fulfill several additional criteria. First of all, they should enhance or facilitate the transfection process during vaccination that may translate to higher antigen expression and immunological response. Secondly, they also should form DNA complexes that encapsulate and protect the DNA vaccines. Their ability to act as adjuvants is also favored. For example, the cationic lipid formulation Vaxfectin® (Vical Inc.) has been shown to effectively enhance immune response after DNA vaccination, not due to increased gene transfer efficiency, but rather by direct modulation of immune pathways [15, 16]. This adjuvant which is a mixture of a cationic lipid (GAP-DMORIE) and a neutral phospholipid (DPyPE) was also tested with protein and inactivated vaccines with promising results.

Recently, PTAI were demonstrated by us to allow effective transfection of plasmid DNA complexes into cells [10, 11]. The aim of this study was evaluation of PTAI as an efficient carrier of DNA vaccines. Several experimental DNA vaccines consisting of K3 plasmid and PTAI compositions with different PTAI : DNA ratios were studied in two animal models, chickens (broiler and layer) and mice. As a reference vaccine we used a mixture of the vaccine plasmid with the transfection reagent Lipofectin®, which is a 1 : 1 (*w*/*v*) liposome formulation of the cationic lipid DOTMA (N-[1-(2,3-dioleyloxy) propyl]-n,n,n-trimethylammonium chloride) and DOPE. Previously, such a vaccine composition has been shown by us to be highly immunogenic and a dose of 125 μg of DNA protected chickens against challenge with homologous and heterologous H5N1 virus [12].

In the first animal experiment we used mixtures containing a high concentration of DNA (125 μg/dose) and variants of PTAI with various length polyprenyl chains mixed with various combinations of helper lipids: DOPE, DOPE/DC or DOPE/DC/DOPC. All compositions gave positive results in ELISA and HI tests; however, a high variability of humoral response was apparent in the K3-125/PTAI-11/DOPE/DC/DOPC and K3-125/PTAI-15/DOPE/DC/DOPC groups, resulting in significantly lower antibody levels than other formulations. Interestingly, in both cases we observed a slow process of precipitation in the vaccine mixture after blending the vaccine plasmid with the PTAI composition, which might be responsible for the observed variability of the individual immune responses. The highest HI titers (as well as the highest median and geometric mean of the HI titer) were in the K3-125/PTAI-11/DOPE/DC group (Fig. 2a and b). Therefore, this PTAI composition was further examined using the lower dose of the plasmid (62.5 μg) in two DNA : PTAI ratios and compared with control carrier and no carrier. The PTAI composition consisting of a PTAI : DNA (*w*/w) ratio of 0.8 : 1 seemed to be more effective in stimulating specific anti-HA antibodies than the controls. Presumably, it was more effective than the control lipid in provoking endocytosis and further DNA release, which might lead to higher production of the target antigen. It is also worth mentioning that we observed some differences between the two chicken experiments, revealing some inconsistencies in the general effectiveness of immunizations in both the PTAI and Lipofectin groups. A possible explanation may be the different chicken lines used (with different genotype and phenotype) and relatively small number of individuals in groups.

Additionally, we decided to use a similar vaccine composition K3/PTAI-10-14/DOPE/DC in mice experiments. From a potential commercial perspective, the administration of a mixture of polyprenols with different length chains instead of a single polyprenol (with a defined length polyprenol chain) is economically attractive. A mixture is easier to obtain due to the lack of a chromatographic separation step after plant material isolation. Thus, the PTAI-10-14 mix could be a simpler and cheaper replacement for the highly effective PTAI-11 lipid. Previously, for the same reasons, the PTAI-6-8 mixture was chosen to be tested in the first chicken experiment. The experiment with mice confirmed that the PTAI-10-14 mixture could be used as an efficient DNA carrier for DNA vaccination purposes.

Efficient delivery of plasmid DNA into host cells is a challenge of great importance for the development of DNA vaccines. Early DNA studies in large animal models and the first human trials resulted in little induction of immune response in comparison to mouse models. A possible explanation may be differences in the DNA uptake of target cells [17]. Intramuscular injection, which is the most widely used way of DNA immunization is very susceptible to inter-individual variation related to differences in cell transfection level at the injection site, as most of the intramuscularly-injected bare DNA does not actually transfect cells and is phagocytosed [18]. This lowers general vaccine efficacy in the population and disrupts the results of clinical trials. Improvements in efficient and less accidental DNA transfer which results in high and more equal expression in cells make DNA vaccines more effective. Besides mechanical devices and physical stimulus, various chemical or biomaterial carriers (like the cationic lipids used in this work) are widely investigated and represent the most promising strategy for DNA vaccination [19].

## Conclusion

In this work, cationic derivatives of polyprenols (PTAI) were tested in vivo as carriers of DNA vaccines. Several variants of formulations containing a mixture of different PTAI with helper lipids were able to induce high immune responses against the target antigen in chickens and mice. Such carriers, which can be obtained from common plant material, are a promising approach for DNA vaccine optimization, especially given that there is a great need for systems that make use of natural materials and processing conditions.

## Abbreviations

DC: DC-cholesterol
HA: Hemagglutinin
PTAI: Cationic derivatives of polyprenols
